# Complex Oncologic Surgery in a Nonagenarian With Severe Aortic Stenosis: Integration of Enhanced Recovery After Surgery Protocol, Total Intravenous Anesthesia, and Advanced Hemodynamic Monitoring

**DOI:** 10.14740/jmc5279

**Published:** 2026-03-27

**Authors:** Alert Drishti, Teuta Dedej-Kurti, Rudin Domi, Gentian Huti, Asead Abdyli, Driola Hoxha, Oliatina Demiri, Besnik Filaj, Diamant Lulaj, Krenar Lilaj, Majlinda Naco, Alma Cani, Hektor Sula, Filadelfo Coniglione, Vedat Eljezi

**Affiliations:** a Department of Surgery, Clinical Toxicology Service, University of Medicine, Tirana, Albania; b Laboratory Department, University of Medicine, Tirana, Albania; c Department of Surgery, Service of Anesthesia and Intensive Care, University of Medicine, Tirana, Albania; d Department of Anesthesiology and Intensive Care, American Hospital 3, Tirana, Albania; e Department of Cardiology, American Hospital 3, Tirana, Albania; f Department of Clinical Science and Translational Medicine, Tor Vergata University of Rome, Rome, Italy; g Department of Perioperative Medicine, CHU Gabriel-Montpied, Clermont-Ferrand, France

**Keywords:** Elderly, Major surgery, Frailty, Aortic stenosis, Total intravenous anesthesia, Enhanced recovery after surgery

## Abstract

The number of elderly patients presenting for major surgery is steadily increasing, and perioperative management in this population remains challenging because of frailty, multiple comorbidities, and reduced physiological reserve. Severe aortic stenosis represents a particularly high-risk condition due to fixed stroke volume and sensitivity to even brief episodes of hypotension or tachycardia. We describe the perioperative course of a 92-year-old woman who underwent combined right radical nephroureterectomy, cystectomy, and total hysterectomy with bilateral salpingo-oophorectomy, using a multimodal strategy that included total intravenous anesthesia, enhanced recovery after surgery principles, and advanced hemodynamic monitoring with MOSTCARE. Despite considerable cardiovascular and surgical risk, intraoperative hemodynamics remained stable, the patient was extubated in the intensive care unit 2 h after surgery, and postoperative recovery was uneventful. This case demonstrates that well-selected very elderly patients may successfully undergo complex oncologic surgery when care is individualized, multidisciplinary collaboration is strong, and modern perioperative strategies are rigorously applied.

## Introduction

The demographic shift toward an aging population has led to a growing number of patients over 80 years of age presenting for major surgery. Historically, outcomes in this group were poor, and many procedures were considered prohibitive. However, improvements in anesthetic techniques, perioperative monitoring, and structured care pathways have progressively changed clinical attitudes. Contemporary evidence suggests that surgical risk is determined more by physiological reserve, comorbid burden, and functional capacity than by chronological age alone [1–3].

Among elderly surgical candidates, severe aortic stenosis (AS) poses one of the greatest anesthetic challenges. These patients depend on adequate preload and afterload to sustain coronary perfusion. They are extremely sensitive to reductions in systemic vascular resistance, arrhythmias, and tachycardia; even transient hypotension can precipitate myocardial ischemia, heart failure, or sudden cardiovascular collapse [4–6]. The physiological stress of prolonged abdominal and pelvic surgery, combined with anesthesia-induced vasodilation, magnifies these risks.

Parallel to these concerns, enhanced recovery after surgery (ERAS) programs have gained wide acceptance as structured, multidisciplinary pathways designed to attenuate surgical stress, maintain physiological function, and shorten convalescence. Although originally applied in younger surgical populations, ERAS has demonstrated benefits in elderly cohorts, including reductions in complications, opioid exposure, and length of stay [7–9]. When combined with individualized goal-directed fluid and hemodynamic therapy, ERAS may mitigate the risks traditionally associated with advanced age.

We report the perioperative strategy employed in a 92-year-old woman undergoing extensive urologic–gynecologic surgery. The case illustrates the integration of ERAS principles, total intravenous anesthesia (TIVA), and advanced hemodynamic monitoring to support safe surgical management despite significant cardiovascular risk.

## Case Report

### Investigations

The patient was a 92-year-old woman who presented with recurrent urogynecology malignancy requiring definitive surgical management. The patient was diagnosed with extensive urothelial carcinoma involving the entire ureter, with invasion of the urinary bladder, uterus, and ovaries. Her medical history was notable for severe AS, long-standing hypertension, type 2 diabetes mellitus, scoliosis, and rheumatoid arthritis. One year earlier she had undergone left nephrectomy and subsequently developed chronic kidney disease with dialysis catheter access. Despite multiple comorbidities, she remained partially independent in daily activities and expressed a clear preference for active oncologic intervention.

### Diagnosis

**Figure 1:**
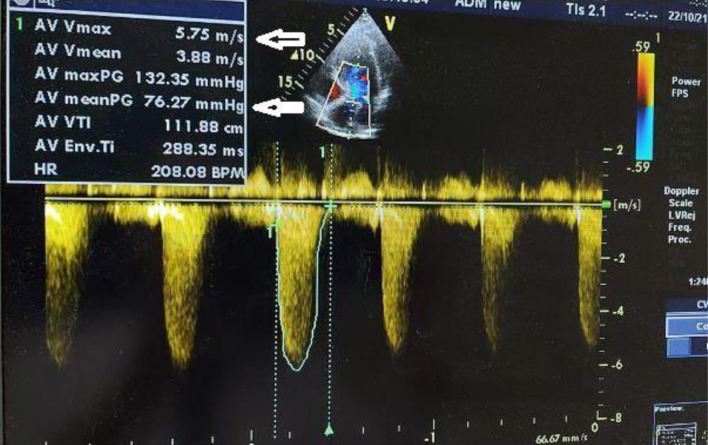
Severe aortic stenosis with a transvalvular mean gradient of 76 mm Hg and maximal velocity of 5.75 m/s (arrows indicates transvalvular mean gradient and maximal velocity).

Preoperative evaluation included transthoracic echocardiography, which confirmed severe AS (with a transvalvular mean gradient of 76 mm Hg, and maximal velocity of 5.75 m/s) with preserved left ventricular systolic function (Fig. 1). Laboratory results showed mild anemia and biochemistries consistent with stable chronic kidney impairment. Cardiologic and anesthetic consultations concluded that although perioperative risk was high, surgical treatment represented a reasonable option given the patient’s goals and expected disease progression. A multidisciplinary conference involving anesthesiology, urology, gynecology, nephrology, and intensive care specialists was convened, and an individualized perioperative plan was developed emphasizing tight hemodynamic control, restrictive yet goal-directed fluid administration, and early postoperative recovery strategies.

### Treatment

Optimization efforts focused on maintaining euvolemia, controlling blood glucose, and ensuring adequate analgesic planning. Medications essential for cardiovascular stability were continued, and detailed discussions with the patient and family addressed risks, benefits, and expected postoperative trajectory. Shared decision-making was central to proceeding with surgery. Subcutaneous enoxaparin (4,000 units) and elastic bandaging were initiated for thromboprophylaxis the day before surgery.

**Figure 2:**
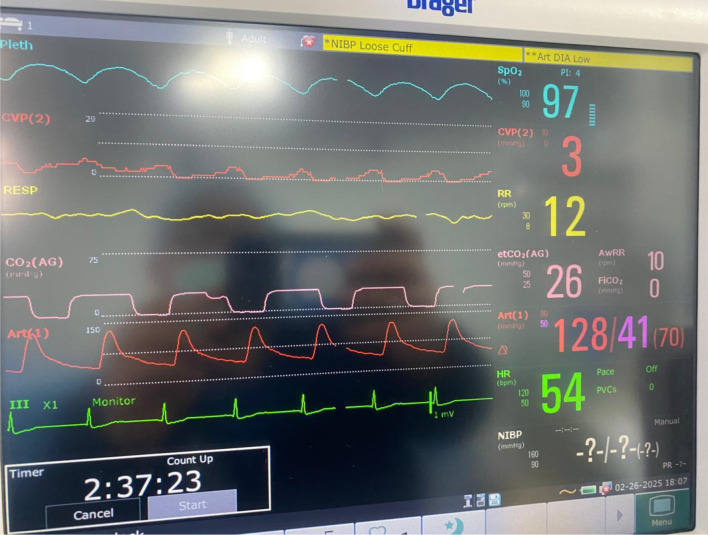
Intraoperative monitoring in accordance with ASA standards, including invasive arterial blood pressure and central venous pressure monitoring.

On the day of surgery, standard American Society of Anesthesiologists (ASA) monitoring was established, and invasive lines were placed to facilitate advanced hemodynamic assessment and vascular access. Cefazolin (1 g) and ondansetron (4 mg) were administered intravenously upon the patient’s arrival in the operating room, where she was continuously monitored. A right radial arterial line was inserted prior to anesthesia induction and endotracheal intubation allowing continuous blood pressure monitoring (Fig. 2). A left internal jugular central venous catheter was inserted to allow administration of vasoactive medications, while the pre-existing femoral dialysis catheter remained available for renal replacement therapy. A nasogastric tube was inserted for gastric decompression.

Anesthetic maintenance was achieved with TIVA. TIVA was selected based on its favorable profile for hemodynamic stability, reduced sympathetic stimulation, and predictable emergence characteristics in elderly patients. The anesthetic strategy prioritized preservation of sinus rhythm, avoidance of tachycardia, maintenance of preload, and prevention of hypotension by supporting systemic vascular resistance when necessary. TIVA was realized using propofol (100–150 µg/kg/min) and remifentanil (0.3–0.5 µg/kg/min), after the anesthesia induction and endotracheal intubation. Noradrenaline was titrated at 0.03–0.08 µg/kg/min to maintain mean arterial pressure (MAP) 70–80 mm Hg and central venous pressure (CVP) 5–8 mm Hg, ensuring stable hemodynamics and adequate tissue perfusion. Fluid therapy followed ERAS-guided restrictive principles, responsive to dynamic indicators of volume status, with 1 L of crystalloid administered. One unit of packed red blood cells was transfused for operative blood loss in the context of pre-existing anemia. Cumulative urine output reached 1 L by the time the remaining kidney was excised.

**Figure 3:**
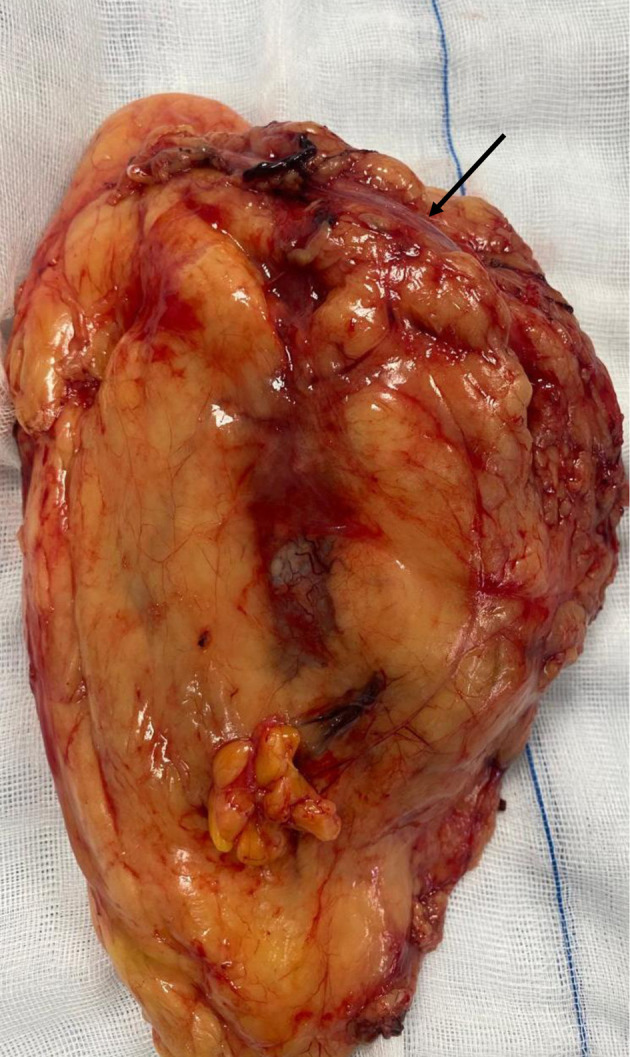
Right radical nephroureterectomy (arrow indicates the ureter).

**Figure 4:**
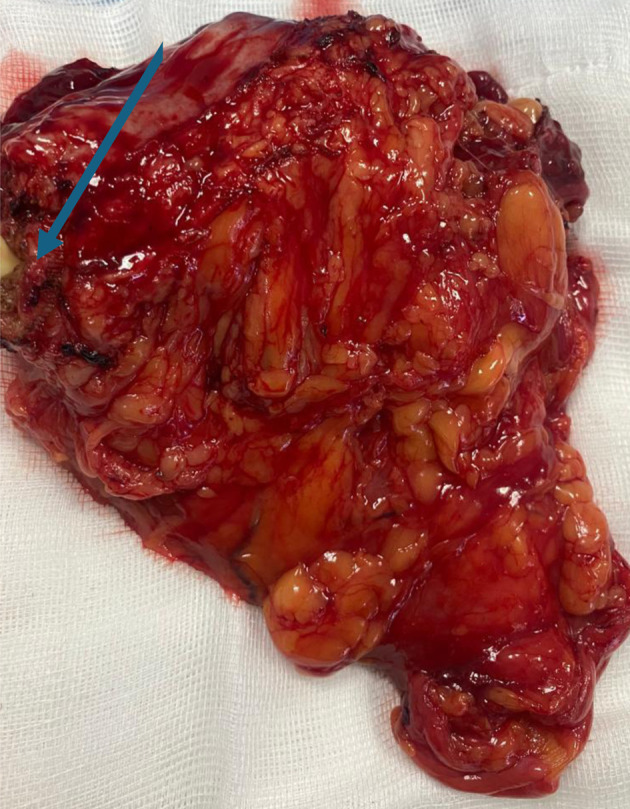
Cystectomy and total hysterectomy with bilateral salpingo-oophorectomy (arrow indicating the urinary bladder neck, and the bladder is visible; the uterus is located beneath the urinary bladder).

The surgical procedure involved a combined laparoscopic and open approach that included right radical nephroureterectomy, cystectomy, and total hysterectomy with bilateral salpinges-oophorectomy. The resected organs were sent in their entirety to the Department of Pathology, without additional dissection, in accordance with departmental protocol (Figs. 3 and 4). The total operative time was approximately 4.5 h. Throughout the procedure, hemodynamics remained stable, and no arrhythmias, ischemic electrocardiographic changes, or significant hypotensive episodes were observed.

### Follow-up and outcome

**Figure 5:**
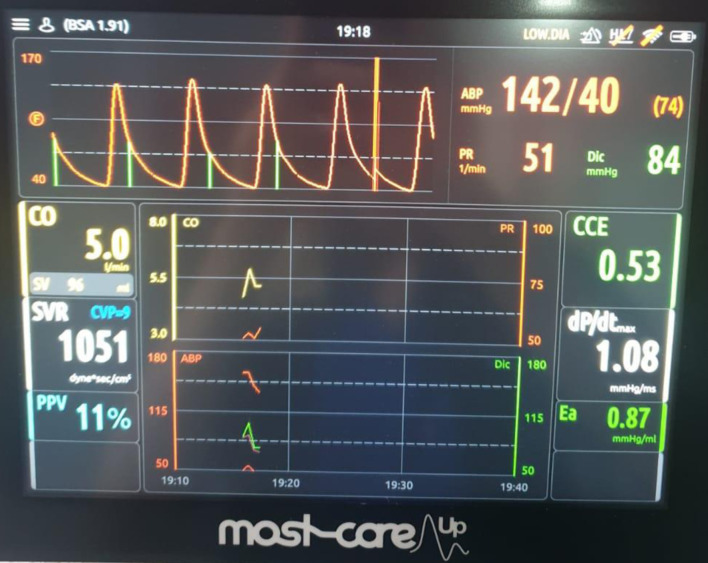
MOST-CARE hemodynamic monitoring during the postoperative period in the intensive care unit, demonstrating cardiac output (CO), stroke volume (SV), systemic vascular resistance (SVR), and pulse pressure variation (PPV) used to guide hemodynamic optimization.

At the end of surgery, the patient was transferred intubated to the intensive care unit (ICU) for planned postoperative observation. Sedation was discontinued, and she was extubated uneventfully 2 h later. Hemodynamics remained stable without increasing vasopressor requirements, and oxygenation was adequate on supplemental nasal oxygen. MOSTCARE pulse contour monitoring (Fig. 5) was initiated to enable continuous assessment of cardiac output and dynamic preload indices, guiding tailored fluid and vasopressor therapy to maintain adequate tissue perfusion while preventing fluid overload in the anephric patient. Daily laboratory testing included serum chemistry to track creatinine trends and determine the need for hemodialysis, cardiac biomarkers to monitor myocardial function, liver function tests, coagulation indices, and hemoglobin level. Arterial blood gases were obtained frequently to evaluate oxygenation, carbon dioxide levels, electrolyte balance, and acid-base status.

Analgesia was provided using a multimodal regimen, including nonsteroidal anti-inflammatory drugs (paracetamol and ibuprofen), infiltration of the surgical ports with bupivacaine, and intraperitoneal instillation of the same local anesthetic. Regular screening for postoperative delirium was performed and remained negative. ERAS milestones were introduced progressively, including early mobilization, respiratory physiotherapy, and advancement of diet as tolerated. The ICU stay was uneventful, and the patient was subsequently transferred to the surgical ward, where recovery and physiotherapy continued without major complication.

### Oncologic considerations

**Figure 6:**
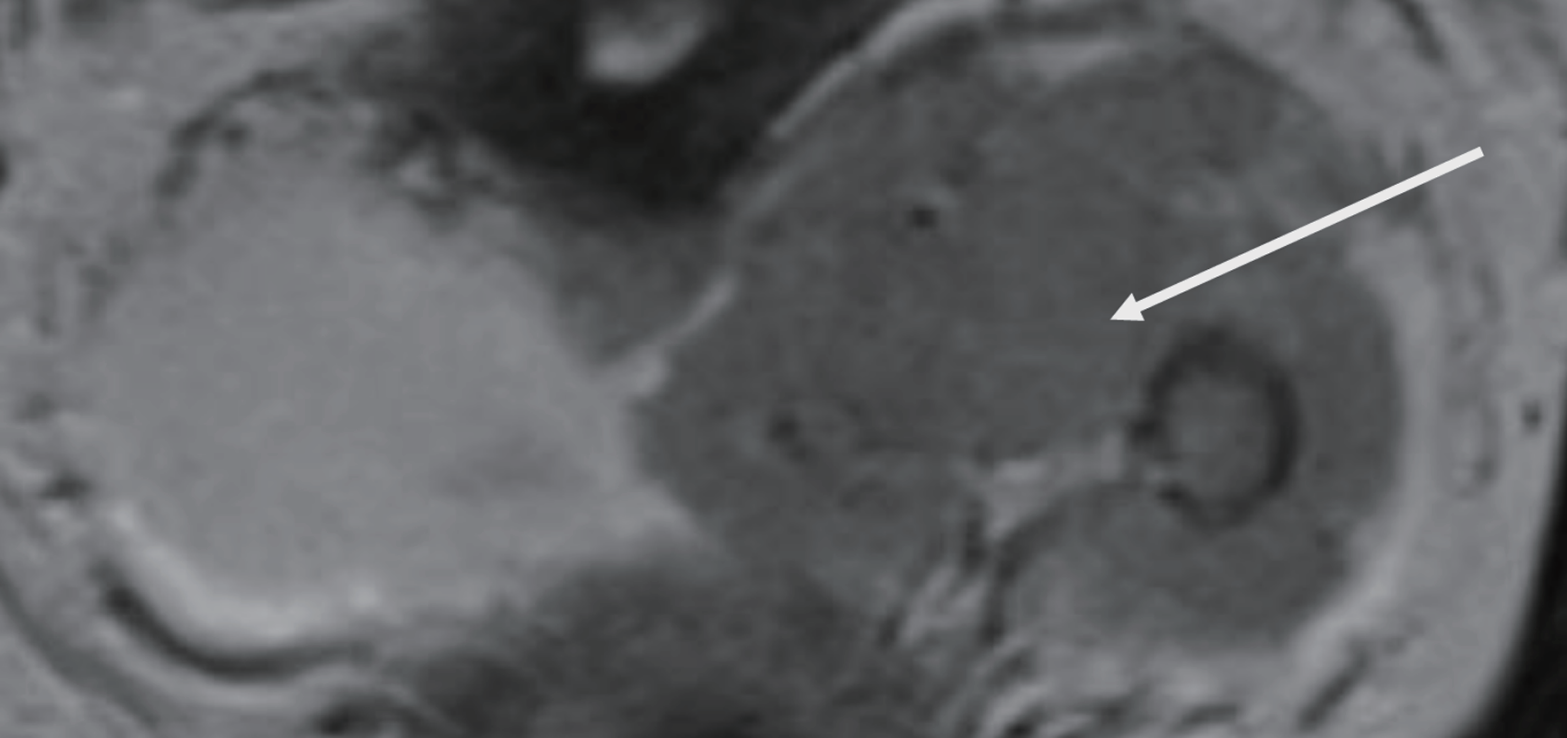
Lower abdomen magnetic resonance imaging (MRI) examination demonstrated tumor invading ureter (arrow).

**Figure 7:**
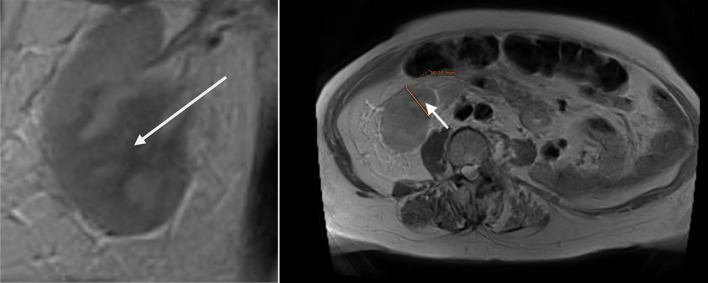
Upper abdomen magnetic resonance imaging (MRI) examination demonstrated tumor invading renal parenchyma (arrow).

**Figure 8:**
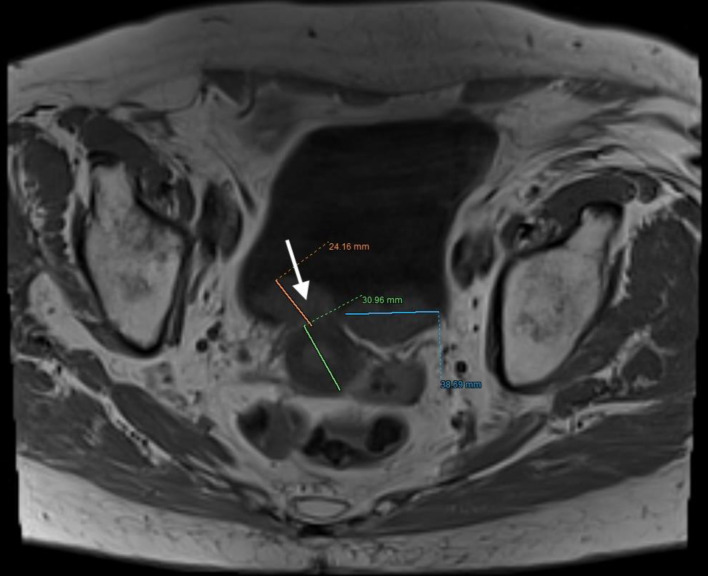
Lower abdominal magnetic resonance imaging (MRI) revealed a vegetative, infiltrative lesion invading the surrounding adipose tissue and occupying the urinary bladder cavity (arrow).

**Figure 9:**
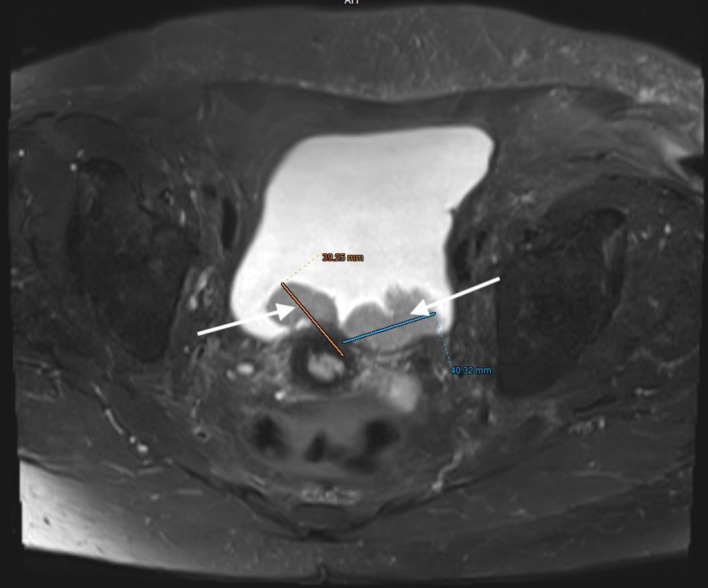
Lower abdominal magnetic resonance imaging (MRI) revealed a vegetative, infiltrative lesion invading the surrounding adipose tissue and occupying the urinary bladder cavity (arrows).

**Figure 10:**
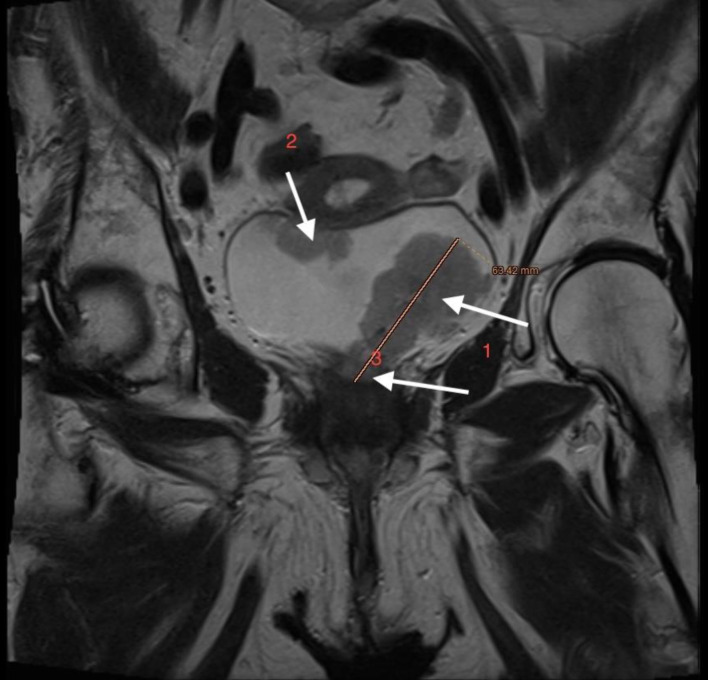
Lower abdominal magnetic resonance imaging (MRI) demonstrated an infiltrative tumor involving the urinary bladder and distal ureter (arrows 1 and 2), with extension into the uterus (arrow 3).

**Figure 11:**
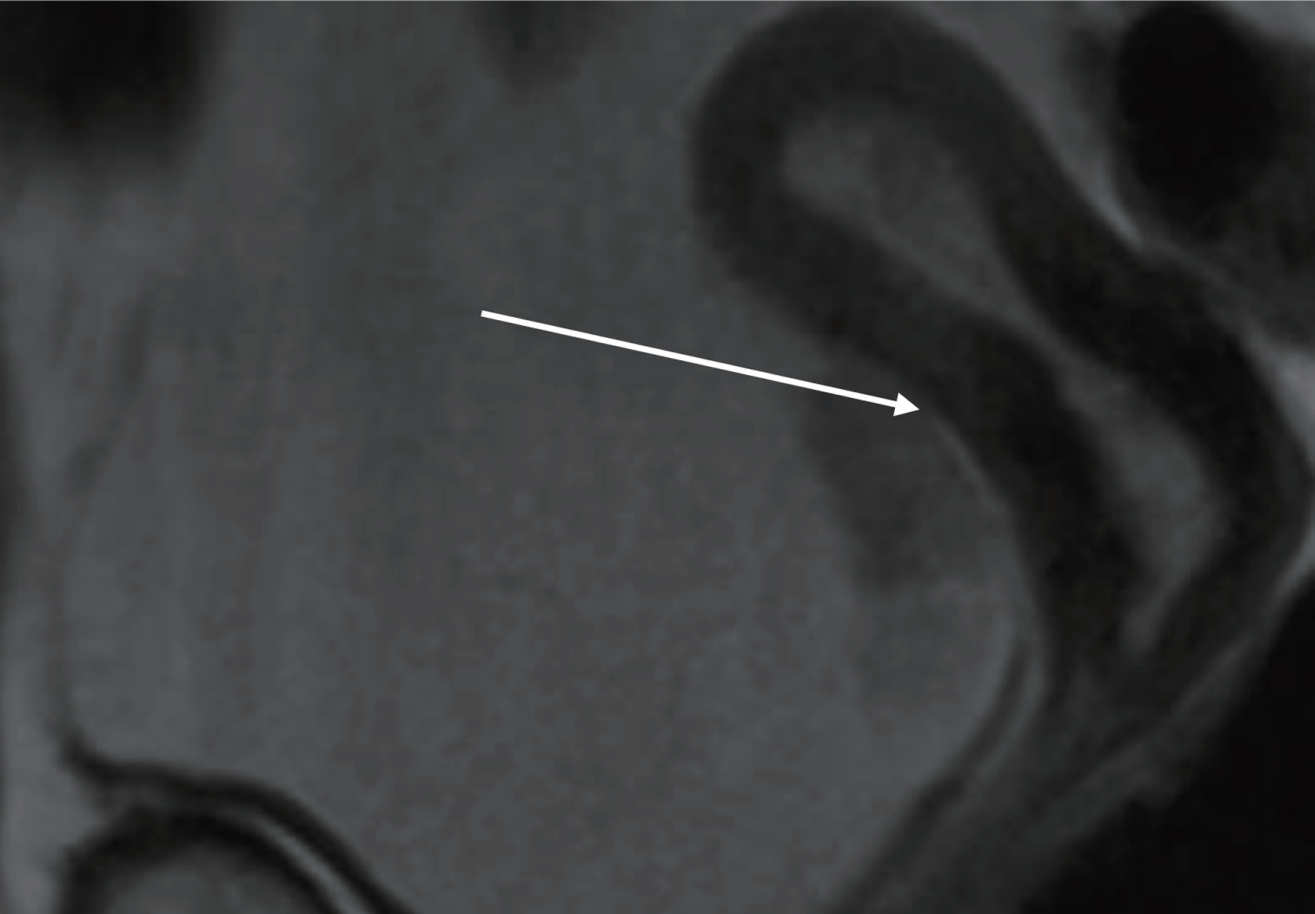
Lower abdominal magnetic resonance imaging (MRI) indicated possible infiltration of uterus (arrow).

The patient, who had undergone a left nephroureterectomy 1 year earlier for urothelial carcinoma, presented again with suspected malignancy of the right upper urinary tract. Following urological consultation and instrumental evaluation (cystoscopy), urinary bladder tumors were detected, and the patient proceeded to further imaging assessment. Magnetic resonance imaging (MRI) of the upper abdomen revealed a tumor in the right kidney with invasion of the ureter and perirenal adipose tissue (Figs. 6 and 7). Lower abdominal MRI examination confirmed urinary bladder tumors with infiltration of the distal ureter and uterus (Figs. 8–11). Considering the patient’s clinical status, the potential aggressiveness of the malignancy, the absence of definitive histopathological confirmation of the new tumors (which could represent distinct lesions requiring separate pathological sampling), and the patient’s preference, no preoperative neoadjuvant therapy was administered. Surgery was therefore the only available therapeutic option, to which the patient provided full informed consent, despite her high-risk clinical profile and the complexity of the proposed high-risk surgical procedure. The postoperative histopathological report demonstrated high-grade renal urothelial carcinoma with contiguous extension to the ureter, urinary bladder, and bladder neck. Five of the 11 excised lymph nodes were metastatic, classifying the tumor as pT4N2Mx. Despite radiological evidence suggestive of uterine involvement, histopathological examination of the uterus and ovaries showed no malignancy; instead, polypoid endometrial hyperplasia and ovarian cysts were identified.

Following discussion by the multidisciplinary oncologic board (including an oncologist, urologist, and radiologist), a comprehensive treatment plan was proposed; however, the patient declined all recommended postoperative adjuvant therapies.

## Discussion

This case highlights how a comprehensive and individualized approach can enable successful completion of complex oncologic surgery in a nonagenarian with significant cardiovascular pathology. The decision to proceed was guided not by chronological age alone, but by an integrated evaluation that included functional status, frailty assessment, comorbidity control, oncologic prognosis, and patient preference. Contemporary data consistently indicate that postoperative outcomes in elderly patients are driven primarily by physiological reserve and frailty rather than age per se, with frailty predicting complications, dependency, and mortality more accurately than chronological age [10–13]. Careful preoperative geriatric assessment therefore represents a critical component of risk stratification in very elderly surgical candidates.

Severe AS introduces major anesthetic challenges due to fixed left-ventricular outflow obstruction and dependence on adequate preload and afterload to maintain coronary perfusion. Even short episodes of hypotension or tachycardia can precipitate myocardial ischemia, pulmonary edema, and circulatory collapse [14–17]. Induction and maintenance of anesthesia must therefore avoid abrupt reductions in systemic vascular resistance and large variations in heart rate. In this patient, maintenance of sinus rhythm, tight blood-pressure control, and avoidance of excessive vasodilation were prioritized. These principles are widely recommended in perioperative cardiovascular guidelines and observational studies involving patients with significant AS [14–17]. The case illustrates how adherence to these hemodynamic goals can mitigate otherwise prohibitive cardiac risk.

Advanced hemodynamic monitoring was central to intraoperative decision-making. Devices based on pulse contour or cardiac-output–guided therapy permit continuous estimation of stroke volume and dynamic preload indices, allowing earlier recognition of inadequate perfusion and tailored titration of fluids and vasoactive drugs. Randomized and consensus data suggest that such goal-directed strategies may reduce complications, shorten hospitalization, and improve tissue perfusion, particularly in high-risk surgical populations [18–21]. In the present case, this technology supported judicious volume administration, avoidance of fluid overload, and maintenance of coronary perfusion pressure.

ERAS principles complemented the hemodynamic strategy by attenuating the metabolic and inflammatory stress response. Multimodal analgesia, early mobilization, limited opioid exposure, gastrointestinal optimization, and restrictive fluid management have been consistently associated with reduced morbidity, fewer complications, and shorter length of stay, effects that extend to elderly cohorts when protocols are carefully individualized [22–25]. Early extubating and absence of delirium in this patient likely reflect the synergistic benefit of these measures. Minimizing functional decline after hospitalization is particularly crucial in older adults, as postoperative immobility and complications strongly predispose to new disability [23–25]. Preoperatively, our patient was managed according to ERAS principles. She participated in structured education and shared decision-making to align expectations and goals of care. Nutritional screening was performed; intravenous iron and erythropoietin were preoperatively administered. A prehabilitation plan was initiated, including respiratory exercises and strategies to facilitate early postoperative mobilization. Her comorbid conditions were carefully optimized, with particular attention to cardiovascular and pulmonary status. Fasting time was minimized in 3 h, and routine bowel preparation was avoided because not indicated. For this patient, intraoperative management was planned and delivered according to ERAS principles. A minimally invasive approach was used for the radical nephroureterectomy, while the remainder of the procedure was performed via an open approach according to the surgeon’s discretion. Active warming was used to maintain normothermia throughout the procedure. Intravenous fluids were administered using a restrictive, goal-directed strategy to balance tissue perfusion while preventing fluid overload. Prophylaxis against postoperative nausea and vomiting was administered, venous thromboembolism prevention was adjusted to her oncologic risk, and antibiotic prophylaxis was provided in line with stewardship recommendations, ensuring correct timing and duration.

TIVA was selected to support stable hemodynamics and predictable emergence. Propofol-based regimens allow precise titration and can reduce sympathetic surges associated with volatile anesthetics, potentially limiting myocardial oxygen demand in patients with critical valvular disease [22–24]. Pharmacokinetic–pharmacodynamic models further facilitate individualized dosing in frail patients with altered drug distribution and clearance. Although randomized comparisons show broadly similar outcomes between volatile anesthesia and TIVA, individualized selection based on cardiac risk and physiological goals remains appropriate [24]. In this patient, TIVA aligned well with ERAS priorities for early awakening and smooth recovery.

Equally important was structured multidisciplinary collaboration. Shared planning among anesthesiology, surgery, cardiology, geriatrics, nephrology, and critical care ensured alignment of therapeutic goals across the perioperative pathway. Vigilant postoperative observation in the ICU allowed rapid treatment of hemodynamic deviations and prevention of complications such as myocardial injury, delirium, and hypothermia, all of which are more frequent in elderly adults and associated with worse outcomes [19–25]. Continuous reassessment and communication were essential components of safety.

Multimodal postoperative pain management was considered an important key point in management of this patient [26]. Pain control was achieved using an opioid-sparing regimen, with bupivacaine administered both by infiltration of the laparoscopic incision sites and by intraperitoneal instillation [27].

### Risk assessment and shared decision-making

Formal risk calculators, including the American College of Surgeons NSQIP Surgical Risk Calculator and the Surgical Mortality Probability Model, can help quantify perioperative risk and facilitate informed discussions with patients and families [21, 22]. In elderly patients with severe cardiac disease, shared decision-making should integrate projected surgical benefit, quality-of-life considerations, and patient goals, alongside predicted complication rates [10, 22]. In the present case, the patient’s strong preference for curative surgery guided the perioperative plan while keeping safety margins strict.

### Transcatheter aortic valve replacement (TAVR) versus surgical considerations

For very elderly patients with severe AS, TAVR may represent an alternative to open cardiac surgery, particularly when combined with non-cardiac surgery [11]. However, in isolated oncologic procedures, definitive valvular intervention may not always be feasible prior to tumor resection. Literature suggests that careful perioperative optimization, hemodynamic monitoring, and anesthetic planning can allow selected high-risk patients to undergo non-cardiac surgery safely even in the presence of critical AS [14–17]. This case illustrates that, when TAVR or staged procedures are not pursued, meticulous multidisciplinary management remains essential.

Taken together, this experience reinforces the principle that advanced age alone should not preclude major surgery. When risk stratification integrates frailty assessment, functional capacity, and patient-centered goals, and when contemporary perioperative strategies such as ERAS protocols, advanced hemodynamic monitoring, and individualized anesthetic management are implemented, the extensive surgical procedures can be performed safely, even in carefully selected nonagenarian patients [10–25].

A multidisciplinary team including urologists, anesthesiologists, intensivists, nephrologists, oncologists, and cardiologists, evaluated the feasibility of performing TAVR, a routine procedure at our institution. However, the patient refused the intervention. In addition, newly emerging clinical factors, such as the increased risk of bacterial endocarditis associated with hemodialysis and an estimated life expectancy of less than 1 year, made TAVR an unsuitable option. Furthermore, patients with a limited life expectancy (< 1 year) or those in whom TAVR is not expected to meaningfully improve quality of life should generally not be considered appropriate candidates for this procedure [28].

### Conclusion

Major oncologic surgery in a 92-year-old woman with severe AS was successfully performed using an integrated approach combining ERAS protocols, TIVA, and advanced hemodynamic monitoring. Careful preoperative planning, vigilant intraoperative control, and structured postoperative care allowed stable hemodynamics, early extubating, and uneventful ICU recovery. This case supports the view that individualized, multidisciplinary perioperative strategies can enable safe surgical management in carefully selected nonagenarian patients, even in the presence of complex cardiac disease.

### Learning points

Very elderly patients should not be excluded from major oncologic surgery solely based on age when physiological reserve and patient goals are carefully assessed.

Advanced hemodynamic monitoring enabled precise titration of fluids and vasopressors, supporting adequate tissue perfusion while minimizing the risk of fluid overload in the anephric patient.

Daily chemistry panels and frequent arterial blood gas analysis provided early warning of renal, cardiac, hepatic, and metabolic derangements, allowing timely intervention.

The combination of restrictive ERAS-guided fluid therapy, close hemodynamic guidance, and multidisciplinary perioperative planning contributed to an uncomplicated postoperative course despite significant cardiovascular risk.

## Data Availability

The authors declare that data supporting the findings of this study are available within the article.
